# Distillery Slops

**Published:** 1882-01

**Authors:** James MacWhinnie

**Affiliations:** Buffalo, N. Y.


### CORRESPONDENCE.

---

Editor Journal of Comparative Medicine and Surgery:—

YOUR well-known interest in all that pertains to the subject of veterinary science prompted the following thoughts on the subject of "Distillery Slops:"

I do not know as I can add anything new concerning our knowledge of this matter, but it has seemed to me that if each of us who practice the profession of comparative medicine would contribute such thoughts and items of interest as come in our way that we might do very much to add to the interest and instruction imparted by your ably conducted journal.

The popular idea seems to be, especially in this region, where "brewers' grains" are commonly used, that they are not the most perfect food for animals, simply the cheapest and most profitable. It is also generally understood that they induce diseased conditions, and that the flesh and milk of animals fed on them is unwholesome and deleterious to the human family. One of our leading papers, the Buffalo *Express*, in a lengthy editorial on the subject of feeding cows "sugar meal" (refuse from the glucose factories) and "distillery slops," asserted that milk produced by cows thus fed was absolutely poisonous to children, as well as detrimental to young animals of their own kind.

Let us see what are the facts in the case. "Distillery slop," when fresh from the still, is only *slightly acid*, provided that the mash has not been allowed to get sour before distillation. If, however, it has been allowed to become very sour, it becomes very rich in lactic and butyric acids, *which acids do cause derangement of the stomach and bowels of animals fed upon it.*

Again, as ordinarily obtained, this slop contains a small proportion of the starch of the grain used in the mash, and also a *notable* quantity of dextrine, which results from the alteration of starch, under the influence of the malt which is used. Dextrine, unlike sugar, cannot be fermented into spirit under the action of yeast.

Dextrine is as nutritive to animals fed upon it as starch, and is much more easily digested. The "distillery slops" also contain, in addition to dextrine and starch, almost all the so-called nitrogenous elements, those which constitute the flesh-forming ingredients of the ordinary grain fed to animals. Theoretically, then, these slops, or "brewers' grains" ought to be wholesome food, when not too sour. But they are not, and this seems to me the reason—*they are too much diluted with water.*

The milk from cows fed too much, or too exclusively on slop, is too thin. The flesh of hogs, horses and cattle thus fed is too watery, and will waste away rapidly, spoil quickly, and the fat of such animals is too soft and oily.

Animals fed on slop exclusively become like inordinate beer-drinkers, bloated, watery and weak, from too much imbibed watery fluid, and the causes of diseased conditions are not as well resisted by them as they would be in a normal state. Slight bruises and scratches in these animals are cured with difficulty, owing to the weakened condition of the tissues. The foregoing facts should be kept in mind and positive directions given that slop should only be fed with a proper proportion of more solid food. Cows, especially, should have an excess of dry hay and other fodder, as a condition of health, if they are kept at all on "brewers' grains" or sugar meal.

Fed in this way, animals retain their health and keep in thriving condition, even when using a considerable preportion of the much-abused "distillery slops."

Respectfully submitted,

JAMES MACWHINNIE, V.S.

BUFFALO, N. Y.

---

EDITOR JOURNAL OF COMPARATIVE MEDICINE AND SURGERY:—

